# Prevalence of Anxiety and Associated Factors among Pharmacy Students in Saudi Arabia: a Cross-Sectional Study

**DOI:** 10.1155/2020/2436538

**Published:** 2020-10-26

**Authors:** Sana Samreen, Nasir A. Siddiqui, Ramzi A. Mothana

**Affiliations:** ^1^Department of Pharmacy, College of Pharmacy, Aurobindo College of Pharmaceutical Sciences Warangal, India; ^2^Department of Pharmacognosy, College of Pharmacy, King Saud University, Riyadh, Saudi Arabia

## Abstract

**Methods:**

We used a cross-sectional design, and data collection was carried out over a period of two months from September 2018 to November 2018 using paper-based self-administered questionnaires. The General Anxiety Disorder-7 (GAD-7) scale was used to measure and classify anxiety among the study participants.

**Results:**

The prevalence of anxiety among pharmacy students was 49% (83 students); 44 students (25.9%) had mild anxiety, while 24 (14.1%) students had moderate anxiety, and 15 (8.8%) severe anxiety. There were statistically significant differences in anxiety scores according to faculty type (*p* = 0.2) and nutritional status (*p* = 0.4).

**Conclusion:**

The findings of this study revealed that half of the pharmacy students suffered from anxiety incidence during their studies at the university. However, the majority of them are experiencing mild to moderate. This may have a significant impact on academic performance and necessitates special attention.

## 1. Introduction

In the context of education and learning, healthcare students can experience high levels of stress in their everyday activities due to a variety of factors such as study burden, high amount of content to be learned in relatively short periods, and continuous exams and tests [1, 2]. This continuous stress can lead to anxiety, nervousness, and worry associated with the arousal of the nervous system [1–4]. Anxiety is a psychological condition as well as an emotional and behavioral disorder characterized by extreme worrying, a sense of fear, agitation, excessively sensitive responses, and deleterious thinking [5, 6]. It was evidenced that anxiety may decrease student's academic interest, due to its physiological symptoms which include sweaty palms, cold hands and feet, panic attacks, fast breathing, racing heartbeat, and upset stomach [3].

Previous studies from different countries and discipline revealed that anxiety and its associated symptoms greatly influence students' academic performance [7–10]. Moreover, several studies reported that most students (75%) during their under graduation and postgraduations at universities and colleges experienced some degree of anxiety symptoms [6, 11]. A large number of studies revealed the association between higher levels of anxiety and poorer academic performance among students [9, 10, 12]. Moreover, earlier data also showed that increased levels of anxiety were associated with decreased memory, loss of concentration, and cognitive decline [13].

Numerous studies reported that the prevalence of anxiety was found to be higher in students compared to the general public [14–18]. The most common form of reported anxiety among the general community was specific phobias with a prevalence rate of 13.2%, followed by social anxiety disorder (5.8%) and generalized anxiety disorder (GAD) (5.1%) [19]. However, the most common prevalent form of anxiety among students was social phobia with 11.9%, showing an early age of onset while panic disorder and GAD had somewhat later onset [20, 21]. Another recent systematic review evaluated the prevalence of depression and anxiety and reported that an estimated prevalence of anxiety was 35% among college students [14]. With regard to Saudi Arabia, recent data indicated that the prevalence of anxiety among graduate and undergraduate medical students was 31.8%; however, the prevalence was higher in first year students compared to those in other years [4]. Another study conducted among undergraduate students in the south region of Saudi Arabia reported a prevalence of 47.2% for mild anxiety, 42.3% for moderate, and 10.5% for severe anxiety [22]. Similarly, a study conducted in the central region of Saudi Arabia with a multiethnic sample of medical students from Al Faisal University reported a high prevalence of 63% of anxiety. To the best of our knowledge, there is scarce data about the prevalence of anxiety among Saudi pharmacy students. Therefore, we aimed this study to assess the prevalence and socioeconomic correlates of anxiety among pharmacy students at Saudi University, Riyadh Saudi Arabia.

## 2. Materials and Methods

### 2.1. Study Design and Participants

The study population consisted of students in the second, third, and fourth year of the Doctor of Pharmacy (PharmD) program and bachelor courses (Bpharm) from King Saud University College of Pharmacy Saudi Arabia. The study excluded junior students, as many of them are not aware of some medical terminology used in the generalized anxiety disorder GAD-7 questionnaire. This was a descriptive cross-sectional study using paper-based questionnaires. Students were randomly invited to participate in the study. We contacted students in their free time before lectures, invited them to participate, and provided them with a summary of the study aims. We started data collection in September 2018 and completed it in the same semester. For the students in each academic year, we picked an exam-free time to gather the data. All participants signed an informed consent form before proceeding for the research.

### 2.2. Study Questionnaire

A structured questionnaire to collect the data for this study was developed according to an extensive review of the study in this field [23]. The questionnaire from the previous study [23] was collected and then redesigned and validated by senior researchers. After validation, a pilot study was initially conducted among senior staff with the help of a researcher at the university school of pharmacy after explaining the study details. Based on the results, the questionnaire was used with some minor modifications. This questionnaire measured pharmacy students' anxiety and was adapted from the GAD-7 [23]. The first section of the questionnaire consisted of demographics and other participant characteristics, including gender, age group, year of study, sleeping habits, source of food, and smoking habits; the second part of the questionnaire included seven items on anxiety parameters (i.e., “Over the last 2 weeks, how often have you been bothered by the following anxiety problems?”) based on the GAD-7.

### 2.3. Prevalence Rate Calculations

The anxiety diagnostic questions were formulated according to the GAD-7, developed by UK clinicians and based on research evidence as well as the UK and European guidelines. The instrument classifies anxiety into three primary types: mild (score of 5-9), moderate (score of 10-14), and severe (>15) anxiety. The GAD-7 score was calculated by assigning scores of 0, 1, 2, and 3, to the response categories of “not at all,” “several days,” “more than half the days,” and “nearly every day,” respectively, and then adding together the scores for the seven questions. Total scores of 5, 10, and 15 were taken as the cutoff points for mild, moderate, and severe anxiety, respectively. When this scale is used as a screening tool, further evaluation is recommended when the score is 10 or greater.

### 2.4. Data Analysis

Manual data entry was carried out from each completed questionnaire received from participants. Data did not include any personal information of respondents such as name or address. A unique identifier associated with the survey questionnaire was used to identify individual responses and analyze the data. The students were informed that their participation was part of a study and voluntary.

Descriptive statistics included frequency counts and percentages and were calculated for each anxiety item. Statistical analyses were performed using SPSS version 25. Mann-Whitney and Kruskal-Wallis tests were applied to explore the association between participant characteristics and anxiety items with a significance level of 0.05. This study was approved by the Research Committee of College of Medicine, King Saud University, Saudi Arabia.

## 3. Results

### 3.1. Participant Characteristics

A total of 170 male pharmacy students from the second, third, and fourth year of bachelor and PharmD programs completed the survey. The majority of the participants (*n* = 120, 76.5%) were aged 21-25 years, and about 23% (*n* = 38) were aged 18-20. One-quarter of the students (*n* = 38, 22%) were enrolled in bachelor's degree programs, while the majority (*n* = 132, 77.6%) were completing the PharmD program (Table 1).

Most of the students were Saudis (*n* = 164, 96%). Of the 170 students who completed the questionnaire, 81 (47.6%) were second year, and 89 (52.3%) were third and 25 (14.7%) fourth year students. Less than a quarter of the students (*n* = 40, 23.5%) were overweight, and 9.4% (*n* = 16) were obese. With regard to lifestyle habits, only 17.6% (*n* = 30) smoked; 45.3% (*n* = 77) ate homemade food, and 40.6% (*n* = 69) restaurant food. A detailed description of participant characteristics is provided in Table 1.

### 3.2. Prevalence of Anxiety

**Figure 1:**
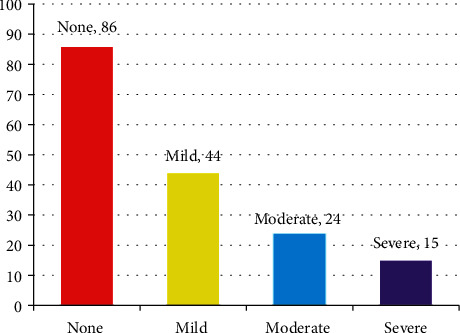
Prevalence of anxiety based on severity.

The prevalence of anxiety among the study participants was 49% (*n* = 86): 44 students (25.9%) had mild anxiety, 24 (14.1%) had moderate anxiety, and 15 (8.8%) had severe anxiety (Figure 1).

### 3.3. Anxiety Parameters

Regarding the symptoms of anxiety, a detailed description of the frequencies of responses to anxiety parameters over the previous 2 weeks is given in Table 2.

As seen in Table 3, there were no differences in total anxiety scores between PharmD (median = 4) and bachelor students (median = 4) (*p* = 0.2); smokers (median = 4) and nonsmokers (median = 4) (*p* = 0.7); between those sleeping less than 6 h/day (median = 3), 6-8 h/day (median = 5.5), and 8-10 h/day (median = 3) (*p* = 0.5); or those in the second (median = 4), third (median = 3.5), and fourth year (median = 5) (*p* = 0.9).

## 4. Discussion

This study identified the incidence of anxiety among pharmacy students from a Saudi university. The total prevalence of anxiety was found to be 49%. A large number of previous studies from different countries utilizing diverse study populations, including both medical and pharmacy students, have investigated the prevalence of anxiety [2, 4, 22, 24]. The prevalence of anxiety in this study was found to be higher in comparison with other studies; for example, a recent study by Ibrahim and Abdelreheem using a sample of (*n* = 164) both medical and pharmaceutical students from Alexandria University reported 29.3% of the prevalence [24]. Similarly, another study from Saudi Arabia estimated the prevalence of anxiety and depression among medical and nonmedical students (*n* = 239) using a cross-sectional design and reported a prevalence of 14% among medical students and 23% among nonmedical students [25]. Another study by Bayram and Bilgel in 2008 among Turkish university students (*n* = 1617) found 47.1% of anxiety [26]. Consistently, another study by Shamsuddin et al. among Malaysian students reported 34% of anxiety [27]. Our study findings were lower than a previous study by Yusoff et al. among medical students (*n* = 442) who reported a high prevalence of anxiety of 64.3% [28], although previous findings suggested that a high prevalence of anxiety (7.7% to 65.5%) among undergraduates were reported in American and European students [29]. However, the prevalence of anxiety may differ among gender, age group, educational year, and academic curriculum [4, 22, 24]. This might be the reason for the high prevalence of anxiety in this current study.

According to previous reports, students from various disciplines such as pharmacy and allied healthcare professions are more likely to be affected by high stress levels related to educational strategies, which results in different levels of consequences in students' academic and social life [22–25]. On the whole, variation in anxiety is expected and can be attributed to the difference in the used study tool and risk factors of anxiety, among which lifestyle (including smoking and student residential status) are critical players.

Recently, there have been important changes in the education curriculum, including the grading system and curriculum [30]. The academic grade will have a great impact on students' ranking system, which will in turn reveal students' status of education and knowledge, affecting their careers [31]. This might be the main factor contributing to the high levels of anxiety and other stress-related problems among students. A number of previous studies found that the prevalence of anxiety was associated with lifestyle factors of students which includes student social status [31–33]. However, two other studies have revealed that females and smokers are more likely to report anxiety [19, 32] in comparison to males and nonsmokers. Additionally, earlier studies also reported that smoking is strongly associated with mental health [31, 33, 34]. On the other hand, it has been reported that increased episodes of anxiety and stress were principal factors leading to an increased smoking prevalence [35, 36]. Moreover, some studies found an association between anxiety and living status, with those living alone reporting more anxiety [31]. However, our study results revealed no significant differences in students' anxiety according to demographic characteristics. Additionally, our results reported significant differences in anxiety according to the course of the study and nutritional status.

Anxiety is regarded as an important factor for students of any discipline, but particularly healthcare, that may negatively influence students' life, both academic and personal. The present results indicated that pharmacy students suffered from some form of anxiety, ranging from mild to severe, reporting that they often felt nervous and were afraid that something awful might happen. Earlier literature on the management of anxiety advised that offering immediate treatment is beneficial to prevent serious consequences [37]. In fact, it was reported that anxiety contributes to depression, which in turn may lead individuals to entertain suicidal thoughts [36, 38, 39]. Furthermore, student counseling is widely used in developed countries such as the USA and the UK to cope with the students' mental and emotional disorders [40]. The British Association for Counseling & Psychotherapy (BACP) has reported the importance of student counseling for achieving advanced knowledge in their curriculum [41]. In Saudi Arabia, previous reports suggested that a student counseling programme was not well recognized like in the USA and the UK. Although studies also reported that public and private universities in Saudi Arabia do have student counselors, but counselors may sometimes lack sufficient or adequate professional qualifications, or lack of adequate experience or understaffed to deal with the students [40, 42]. Undoubtedly, this is a serious issue not only for students but also for society, and further studies are needed to answer these questions.

Our study has several potential limitations. First, it included only second, third, and fourth year pharmacy students; secondly, this study was carried out in a single university in Saudi Arabia; the results may not completely reflect the anxiety of all pharmacy students. The generalizability of our findings should thus be evaluated in future studies. Additionally, we recommend the importance of assessment of anxiety and other behavioral and mental illness using noninvasive tools like questionnaires. This type of assessment may help the policymakers to assess the problems of society timely and make necessary recommendations.

## 5. Conclusion

The findings of this study revealed that half of the pharmacy students suffered from anxiety during their studies at university, with the majority of them experiencing mild to moderate. In addition, our study found no significant differences in anxiety according to participant characteristics. The high burden of studying along with repeated exams are likely to be the major reasons for anxiety. Students' psychological problems such as anxiety, if recognized in early stages, can be treated with behavioral therapy, emotional support, and social skills training. This may help future graduates to overcome their difficulties and lead a healthier life. The present study results highlight the need for further research including larger groups of students in different fields.

## Figures and Tables

**Table 1:** Demographic and basic information of study participants (*N* = 170).

Variables	Frequency (*n*)	Percentage (%)
Age		
18-20	38	22.4
21-25	120	76.5
25-30	2	1.2
Degree type		
PharmD	132	77.6
Bachelor's degree	38	22.4
Year of study		
Second year	81	47.6
Third year	64	37.6
Fourth year	25	14.7
Nationality		
Saudi	164	96.5
Non-Saudi	6	3.5
Nutritional status (body mass index)		
Normal (18.5 to <25)	105	61.5
Overweight (25.0 to <30)	40	23.5
Obese (30.0<)	16	9.4
Moderately obese (30 to <35)	9	5.3
Sleeping pattern		
Less than 6 h/day	68	40
6-8 h/day	82	48.2
8-10 h/day	20	11.8
Smoking		
Yes	30	17.6
No	139	89.8
Source of food		
Homemade	77	45.3
Restaurant	69	40.6
Both	24	14.1

**Table 2:** Anxiety parameters (i.e., “Over the last 2 weeks, how often have you been bothered by any of the following problems”) (*N* = 170).

Anxiety parameters	Frequency	%
Feeling nervous, anxious, or on edge		
Not at all	76	44.7
Several days	55	32.4
More than half the days	16	9.4
Nearly every day	23	13.5
Not being able to stop or control worrying		
Not at all	95	54.1
Several days	45	26.5
More than half the days	13	7.6
Nearly every day	20	11.8
Worrying too much about different things		
Not at all	73	42.3
Several days	50	29.4
More than half the days	22	12.9
Nearly every day	25	14.7
Trouble relaxing		
Not at all	86	50.6
Several days	39	22.9
More than half the days	29	17.1
Nearly every day	16	9.4
Being so restless that it is hard to sit still		
Not at all	105	61.8
Several days	39	22.9
More than half the days	16	9.4
Nearly every day	9	5.3
Becoming easily annoyed or irritable		
Not at all	92	54.1
Several days	44	25.9
More than half the days	15	8.8
Nearly every day	19	11.2
Feeling afraid as if something awful might happen		
Not at all	94	55.3
Several days	38	22.4
More than half the days	18	10.6
Nearly every day	20	11.8

**Table 3:** Associations between total anxiety scores and participant characteristics.

Variables	Mean (median) anxiety score	(std)	*p* value
Faculty type			
cPharmD	5.4 (4)	5.3	0.2^∗^
Bachelor	6.5 (4)	5.9	
Smoking habits			
Yes	4.8 (4)	4.2	0.7^∗^
No	5.8 (4)	5.7	
Sleeping pattern			
Less than 6 h/day	5.1 (3)	5.8	
6-8 h/day	6.5 (5.5)	5.3	0.5^∗∗^
8-10 h/day	4.1 (3)	4.6	
Nutritional status			
Normal	5.5 (4)	4.6	
Overweight	6.5 (4.5)	6.1	0.4^∗∗^
Obese	8.7 (8)	8.07	
Moderately obese	4.2 (4)	3.3	
Year of study			
Second year	5.6 (4)	5.3	
Third year	5.8 (3.5)	5.89	0.9^∗∗^
Fourth year	5.4 (5)	4.55	

^∗^Mann-Whitney test. ^∗∗^Kruskall-Wallis test.

## Data Availability

The data will be available from the corresponding author upon the request.

## References

[1] Bieling P. J., Antony M. M., Swinson R. P. (1998). The state-trait anxiety inventory, trait version: structure and content re-examined. *Behaviour Research and Therapy*.

[2] Abdel Rahman A. G., Al Hashim B. N., Al Hiji N. K., AlAbbad Z. (2013). Stress among medical Saudi students at College of Medicine, King Faisal University. *Journal of Preventive Medicine and Hygiene*.

[3] Vitasari P., Wahab M. N. A., Othman A., Herawan T., Sinnadurai S. K. (2010). The relationship between study anxiety and academic performance among engineering students. *Procedia-Social and Behavioral Sciences*.

[4] Alzahrani A. H., Alzahrani A. K., Alharithy M. (2017). Prevalence of anxiety and generalized anxiety disorder among male medical students at Taif University, Saudi Arabia. *World Journal of Pharmaceutical Research*.

[5] Callahan R. J. (2001). The impact of thought field therapy on heart rate variability (HRV). *Journal of Clinical Psychology*.

[6] Mohd Ghani A., Nubli M., Wahab A., Ahmad O., Prima V. (2010). The use of study anxiety intervention in reducing anxiety to improve academic performance among university students. *International Journal of Psychologica*.

[7] Pillay N., Ramlall S., Burns J. K. (2016). Spirituality, depression and quality of life in medical students in KwaZulu-Natal. *South African Journal of Psychiatry*.

[8] McCraty R., Tomasino D., Atkinson M., Aasen P., Thurik S. J. (2000). *Improving test-taking skills and academic performance in high school students using HeartMath learning enhancement tools*.

[9] McCraty R. (2007). *When Anxiety Causes Your Brain to Jam, Use Your Heart*.

[10] Vogel H. L., Collins A. L. (2006). The relationship between test anxiety and academic performance. *Journal of Abnormal and Social Psychology*.

[11] Tartakovsky M. (2008). Depression and anxiety among college students. *Psych Central*.

[12] Luigi M., Ducci F., Scoto M. C., Passaniti E., D'Arrigo V. G., Vitiello B. (2007). The role of anxiety symptoms in school performance in a community sample of children and adolescents. *BMC Public Health*.

[13] Cassady J. C. (2004). The influence of cognitive test anxiety across the learning–testing cycle. *Learning and Instruction*.

[14] January J., Madhombiro M., Chipamaunga S., Ray S., Chingono A., Abas M. (2018). Prevalence of depression and anxiety among undergraduate university students in low- and middle-income countries: a systematic review protocol. *Systematic Reviews*.

[15] Dyrbye L. N., Thomas M. R., Shanafelt T. D. (2006). Systematic review of depression, anxiety, and other indicators of psychological distress among U.S. and Canadian medical students. *Academic Medicine*.

[16] Oppong Asante K., Andoh-Arthur J. (2015). Prevalence and determinants of depressive symptoms among university students in Ghana. *Journal of Affective Disorders*.

[17] Othieno C. J., Okoth R., Peltzer K., Pengpid S., Malla L. O. (2015). Risky HIV sexual behaviour and depression among University of Nairobi students. *Annals of general psychiatry*.

[18] Ovuga E., Boardman J., Wasserman D. (2006). Undergraduate student mental health at Makerere University, Uganda. *World Psychiatry*.

[19] Baldwin D. S., Anderson I. M., Nutt D. J. (2016). Evidence-based guidelines for the pharmacological treatment of anxiety disorders: recommendations from the British Association for Psychopharmacology. *Journal of Psychopharmacology*.

[20] Blanco C., Okuda M., Wright C. (2008). Mental health of college students and their non-college-attending peers: results from the National Epidemiologic Study on Alcohol and Related Conditions. *Archives of General Psychiatry*.

[21] Kessler R. C., Berglund P., Demler O., Jin R., Merikangas K. R., Walters E. E. (2005). Lifetime prevalence and age-of-onset distributions of DSM-IV disorders in the National Comorbidity Survey Replication. *Archives of General Psychiatry*.

[22] Hakami R. M., Mahfouz M. S., Adawi A. M. (2018). Social anxiety disorder and its impact in undergraduate students at Jazan University, Saudi Arabia. *Mental illness*.

[23] Spitzer R. L., Kroenke K., Williams J. B., Löwe B. (2006). A brief measure for assessing generalized anxiety disorder: the GAD-7. *Archives of Internal Medicine*.

[24] Ibrahim M. B., Abdelreheem M. H. (2015). Prevalence of anxiety and depression among medical and pharmaceutical students in Alexandria University. *Alexandria Journal of Medicine*.

[25] Alattas A. M., Alkhalawi M. J. (2017). The Prevalence of Depression and Anxiety among Medical Students in Comparison with Non-Medical Students: A Cross-Sectional Study in Taibah University, Al Madinah Al Munawwarah, Saudi Arabia. *Prevalence*.

[26] Bayram N., Bilgel N. (2008). The prevalence and socio-demographic correlations of depression, anxiety and stress among a group of university students. *Social Psychiatry and Psychiatric Epidemiology*.

[27] Shamsuddin K., Fadzil F., Ismail W. S. (2013). Correlates of depression, anxiety and stress among Malaysian university students. *Asian Journal of Psychiatry*.

[28] Yusoff M. S., Abdul Rahim A. F., Baba A. A., Ismail S. B., Mat Pa M. N., Esa A. R. (2017). Prevalence and associated factors of stress, anxiety and depression among medical Fayoum University students. *Alexandria Journal of medicine*.

[29] Quek T. T., Tam W. W., Tran B. X. (2019). The global prevalence of anxiety among medical students: a meta-analysis. *International journal of environmental research and public health*.

[30] Al-Arifi M. N., AlDhawailie A., Aldohyan M. (2015). A survey on pharmacist opinion about pharmaceutical care in Saudi Arabia. *Asian Journal of Pharmaceutics*.

[31] Kulsoom B., Afsar N. A. (2015). Stress, anxiety, and depression among medical students in a multiethnic setting. *Neuropsychiatric Disease and Treatment*.

[32] Franko D. L., Striegel-Moore R. H., Bean J. (2005). Self-reported symptoms of depression in late adolescence to early adulthood: a comparison of African-American and Caucasian females. *The Journal of Adolescent Health*.

[33] Wade T. J., Cairney J., Pevalin D. J. (2002). Emergence of gender differences in depression during adolescence: national panel results from three countries. *Journal of the American Academy of Child and Adolescent Psychiatry*.

[34] Whalen C. K., Jamner L. D., Henker B., Delfino R. J. (2001). Smoking and moods in adolescents with depressive and aggressive dispositions: evidence from surveys and electronic diaries. *Health Psychology*.

[35] Trosclair A., Dube S. R. (2010). Smoking among adults reporting lifetime depression, anxiety, anxiety with depression, and major depressive episode, United States, 2005-2006. *Addictive Behaviors*.

[36] Pesko M. F. (2014). Stress and smoking: associations with terrorism and causal impact. *Contemporary Economic Policy*.

[37] Flannery-Schroeder E. C. (2006). Reducing anxiety to prevent depression. *American Journal of Preventive Medicine*.

[38] Carter R. M., Wittchen H. U., Pfister H., Kessler R. C. (2001). One-year prevalence of subthreshold and threshold DSM-IV generalized anxiety disorder in a nationally representative sample. *Depression and Anxiety*.

[39] Wittchen H.-U., Zhao S., Kessler R. C., Eaton W. W. (1994). DSM-III-R generalized anxiety disorder in the National Comorbidity Survey. *Archives of General Psychiatry*.

[40] Alotaibi T. (2015). Combating anxiety and depression among school children and adolescents through student counselling in Saudi Arabia. *Procedia-Social and Behavioral Sciences*.

[41] BACP (2013). *School based counselling. what it is and why we need it. (May), British Association for Counselling & Psychotherapy*.

[42] Alotaibi T. (2014). Challenging existing views of the role of school counsellors in the Kingdom of Saudi Arabia. *8th Ann ua lKeele Counselling Psychology Conference (Keele University, 'Daring to Make an Impact: Dynamic Qualitative Research', 22- 23 March)*.

